# The diagnostic performance of the YEARS and Pulmonary Embolism Graduated D-dimer algorithms in patients with prior venous thrombosis suspected of pulmonary embolism

**DOI:** 10.1016/j.rpth.2026.106617

**Published:** 2026-04-30

**Authors:** Emily S.L. Martens, Vicky Mai, Veronica Bates, Aurelian Delluc, Philippe Girard, Menno V. Huisman, Susan R. Kahn, Clive Kearon, Michael J. Kovacs, Amanda Pecarskie, Marc Righini, Marc Rodger, Dimitrios Scarvelis, Sam Schulman, Sudeep Shivakumar, Melanie Tan, Venkatesh Thiruganasambandamoorthy, Shaun Visser, Philip S. Wells, Grégoire Le Gal, Frederikus A. Klok

**Affiliations:** 1 Division of Thrombosis and Hemostasis, Department of Medicine, Leiden University Medical Center, Leiden, Netherlands; 2 Department of Medicine, Ottawa Hospital Research Institute, University of Ottawa, Ottawa, Canada; 3 Département de Pneumologie, Institut Mutualiste Montsouris, Paris, France; 4 French Clinical Research Infrastructure Network INvestigation Network On Venous Thrombo-Embolism Network, Saint-Etienne, France; 5 Division of Internal Medicine, Department of Medicine, McGill University, Montreal, Quebec, Canada; 6 Department of Medicine and Thrombosis and Atherosclerosis Research Institute, McMaster University, Hamilton, Ontario, Canada; 7 Division of Hematology, Department of Medicine, University of Western Ontario, London, Ontario, Canada; 8 Division of Angiology and Hemostasis, Geneva University Hospitals and Faculty of Medicine, Geneva, Switzerland; 9 Department of Medicine, McGill University, McGill University Health Center, Montreal, Quebec, Canada; 10 Department of Medicine, Dalhousie University, Nova Scotia Health, Halifax, Nova Scotia, Canada; 11 Department of Medicine, Hospital Gelderse Vallei, Ede, Netherlands; 12 Department of Emergency Medicine, University of Ottawa, Ottawa, Ontario, Canada; 13 Department of Emergency Medicine, Hôpital Montfort, Ottawa, Ontario, Canada

**Keywords:** clinical decision rules, diagnosis, pulmonary embolism, venous thrombosis

## Abstract

**Background:**

Evidence on how diagnostic algorithms perform in patients with prior venous thromboembolism (VTE) is scarce.

**Objectives:**

This study determined the diagnostic miss rate during 3-month follow-up and the proportion of avoided imaging using the YEARS and Pulmonary Embolism Graduated D-dimer (PEGeD) algorithms.

**Methods:**

This preplanned analysis was part of the international prospective multicenter PREDICTORS study that included outpatients suspected of recurrent VTE managed with validated diagnostic algorithms and D-dimer assays per local practices. Clinical variables were collected to compute the original Wells, modified Wells, and revised Geneva scores. Patients without VTE were followed up for 3 months. Events were independently adjudicated. For this analysis, YEARS and PEGeD scores were computed post hoc for patients with suspected pulmonary embolism (PE).

**Results:**

In total, 188 patients were included. Overall, PE incidence at baseline was 31% (58/188), and 47% (47/99) in those without anticoagulation. Applying YEARS, 87 patients (46%) would have had PE ruled out without imaging. The overall diagnostic miss rate would have been 2 of 83 (2.4%; 95% CI, 0.7%-8.4%) and 2 of 29 (6.9%; 95% CI, 1.9%-22%) in those without anticoagulation: both patients had no YEARS items and D-dimer of <1000 ng/mL but had imaging at baseline showing PE. For PEGeD, these numbers were 80 (43%) for efficiency, with an associated diagnostic miss rate of 2 of 77 (2.6%; 95% CI, 0.7%-9.0%) and 2 of 26 (7.7%; 95% CI, 2.1%-24%) in those without anticoagulation: both patients had a Wells score of <4.5 and D-dimer of <1000 ng/mL but had imaging at baseline showing PE.

**Conclusion:**

Both diagnostic algorithms showed comparable safety and efficiency in patients with prior VTE. Notably, the diagnostic miss rate was higher than expected and exceeded the safety margin.

## Essentials

• The accuracy of diagnostic algorithms for recurrent venous thromboembolism (VTE) is uncertain.
• This is an analysis of the prospective PREDICTORS study in patients with previous VTE.
• The YEARS and PEGeD algorithm demonstrated similar safety and efficiency profiles.
• Imaging was avoided in 43%, with high diagnostic miss rates exceeding safety thresholds.

## 1 Introduction

Computed tomographic pulmonary angiography (CTPA) is the diagnostic standard for diagnosing acute pulmonary embolism (PE) [1]. While CTPA has many benefits, it is also associated with risks such as radiation exposure, contrast-induced complications (eg, allergy and nephropathy), increased health care utilization, and overdiagnosis of isolated subsegmental emboli with uncertain relevance, as well as misclassification of chronic thrombi [[2], [3], [4], [5], [6]]. To help avoid unnecessary and potentially harmful diagnostic imaging in patients suspected of having acute PE, several clinical decision rules (CDRs) have been developed that combine a standardized assessment of clinical pretest probability (C-PTP) with D-dimer testing to help identify patients at low risk for PE [[7], [8], [9], [10], [11], [12], [13], [14]].

Diagnostic strategies that use D-dimer thresholds dependent on C-PTP, such as the YEARS and Pulmonary Embolism Graduated D-dimer (PEGeD) algorithm, have been shown to be most efficient when compared with other CDRs in ruling out a first PE [15,16]. However, the diagnostic performance of these algorithms is uncertain in patients presenting with suspected recurrent PE, as they have been scarcely studied specifically in this context [17]. The main potential limitations of these diagnostic algorithms in the setting of recurrent PE are that the specificity of the CDR decreases because of points assigned to a history of venous thromboembolism (VTE) and that the sensitivity of the D-dimer test may decrease, particularly if patients are still using anticoagulation [15,18].

A better understanding of the diagnostic performance of CDRs for suspected PE in this patient population is crucial for optimizing the relevant diagnostic pathways. Therefore, we aimed to assess the diagnostic performance of the YEARS algorithm and the PEGeD algorithm in patients with a history of VTE suspected of having acute PE.

## 2 Methods

### 2.1 Study design and study participants

We conducted a preplanned analysis of the data from the PREDICTORS study (NCT02297373): a prospective, international, multicenter, observational cohort study, aiming at validating existing diagnostic algorithms for VTE in patients suspected of having acute recurrent VTE [19]. In this study, all adult outpatients with a history of VTE, presenting to the emergency departments or thrombosis clinics of participating hospitals with signs or symptoms suggestive of recurrent VTE between November 2014 and January 2019, were managed with validated CDRs and D-dimer assays according to local practices. Exclusion criteria involved the following: (i) presence of medical or co-morbid conditions that made it unlikely that the patient would complete 3 months of follow-up; (ii) suspicion of upper extremity thrombosis or thrombosis at an unusual site (eg, cerebral or splanchnic venous thrombosis); (iii) previous VTE was a distal deep vein thrombosis (DVT) or subsegmental PE.

Clinical variables to compute the original Wells [20], modified Wells [21], and revised Geneva scores [12] were collected for each individual, along with a blood sample for D-dimer testing at the time of the suspected recurrence. D-dimer testing was performed locally at each participating center, including various assays. The diagnostic workup of each study participant was according to the standards of care and national guidelines throughout the duration of the study and at the discretion of the treating physician. All patients in whom a diagnosis of recurrent VTE was ruled out (with or without additional imaging) received a follow-up phone call from study personnel 3 months (90 ±7 days) after the initial testing. During follow-up, a standardized case report form was completed. The patients were advised to contact the study team in case of signs or symptoms of VTE recurrence during follow-up. Patients in whom a diagnosis of VTE was confirmed by imaging at the initial evaluation were treated as per standards of care at the local institution. These patients did not receive a follow-up call. All suspected cases of VTE and deaths were adjudicated by an independent committee. Recurrent PE was diagnosed if V/Q scan was nonnormal with a new unmatched segmental or greater perfusion defect or if CTPA showed a new intraluminal defect in a segmental or greater vessel. Recurrent DVT was confirmed if compression ultrasound revealed a new area of noncompressibility of a venous segment above the trifurcation of the popliteal vein or magnetic resonance direct thrombus imaging showing fresh DVT. Death related to PE was categorized as being certain (eg, hypotension, hypoxia, cardiac arrest with no other explanation than PE, autopsy, or radiographic confirmation), highly probable (criteria for certain but another disease could have caused the death), probable (other cause suspected based on clinical evidence but 100% certainty not available), and unlikely (all other cases) [19]. For the current analysis, only patients suspected of acute PE—either isolated PE or PE with concomitant DVT—at baseline were included.

### 2.2 Diagnostic strategies

The YEARS algorithm has been outlined in the original study article [8] and is depicted in Supplementary Figure 1. In summary, patients with clinically suspected PE are evaluated based on 3 clinical criteria: clinical signs of DVT, hemoptysis, and PE most likely diagnosis. If zero clinical criteria are met (ie, low C-PTP), a D-dimer level of <1000 ng/mL allows ruling out PE without imaging (ie, negative YEARS strategy), whereas a D-dimer level of ≥1000 ng/mL results in the need for imaging (ie, positive YEARS strategy). If 1 or more of the clinical criteria are met (ie, high C-PTP), a D-dimer level of <500 ng/mL will rule out PE without imaging (ie, negative YEARS strategy), whereas a D-dimer level of ≥500 ng/mL results in the need for imaging (ie, positive YEARS strategy).

The PEGeD algorithm has been outlined in the original study article [9] and is depicted in Supplementary Figure 2. In summary, patients with clinically suspected PE are evaluated based on the 7-item Wells score: clinically suspected DVT (3 points), alternative diagnosis is less likely than PE (3 points), heart rate above 100 beats per minute (1.5 points), a history of VTE (1.5 points), hemoptysis (1 point), and cancer or treatment for cancer within 6 months (1 point). If 0 to 4 points are met (ie, low C-PTP), a D-dimer level of <1000 ng/mL will rule out PE without imaging (ie, negative PEGeD strategy), whereas a D-dimer level of ≥1000 ng/mL results in the need for imaging (ie, positive PEGeD strategy). If 4.5 to 6 points are met (ie, moderate C-PTP), a D-dimer level of <500 ng/mL allows ruling out PE without imaging (ie, negative PEGeD strategy), whereas a D-dimer level of ≥500 ng/mL results in the need for imaging (ie, positive PEGeD strategy). A Wells score of ≥6.5 (ie, high C-PTP) requires direct referral for imaging independent of D-dimer test results.

For the current analysis, YEARS and PEGeD scores were computed post hoc for all patients and only VIDAS D-dimer results were used. This was because the standard-of-care D-dimer assays provided only binary results (positive or negative) in a large number of patients, rather than continuous D-dimer levels.

### 2.3 Outcome

The primary outcome was the diagnostic performance of each diagnostic strategy (ie, YEARS and PEGeD algorithm) in ruling out VTE in patients with suspected PE and prior VTE, with or without suspected concomitant DVT of the lower extremities. Measures of diagnostic performance included the diagnostic miss rate, efficiency, and the predictive performance of each diagnostic strategy in ruling out PE at initial testing, expressed in terms of sensitivity, specificity, and positive predictive value (PPV) and negative predictive value (NPV). The diagnostic miss rate—an indicator of safety—was defined as the proportion of patients with confirmed PE at baseline or VTE within the 3-month follow-up period who had been classified as PE excluded by each diagnostic strategy (Supplementary Figures 1 and 2). To prevent overestimation of the safety of the algorithms, patients lost to follow-up were excluded from the safety analysis. The efficiency of a strategy was defined as the proportion of patients classified as PE excluded without imaging by the respective diagnostic strategy.

### 2.4 Statistical analysis

Patient characteristics were described using standard descriptive statistics. Categorical variables are presented as counts with corresponding percentages, while continuous variables are reported as medians with corresponding IQRs in the case of skewed data.

Using the prospectively collected items of the different CDRs, patients were categorized into 2 C-PTP groups (ie, low vs high) according to the YEARS algorithm and into 3 C-PTP groups (ie, low, moderate, and high) according to the PEGeD algorithm. Depending on the C-PTP group, D-dimer results were categorized as negative or positive for each diagnostic strategy.

The incidence of PE at initial testing, as well as the incidence of VTE during the 3-month follow-up period, was calculated for the total cohort and for each subgroup of patients with and without an indication for imaging according to the YEARS and PEGeD algorithm, respectively. The safety and efficiency based on each diagnostic strategy are presented as proportions with corresponding 95% CIs and stratified by anticoagulation status at initial testing. Additionally, the predictive performance of each diagnostic strategy for diagnosing PE was measured in terms of sensitivity, specificity, and predictive values (PPV and NPV) and presented as proportions with corresponding 95% CI. No comparative statistical analysis methods were conducted, as this was beyond the scope of the secondary analysis. Statistical analyses were carried out in SPSS Statistics version 29.0 (IBM) [22].

## 3 Results

### 3.1 Patient characteristics

Of the 723 patients enrolled in the PREDICTORS study [19], 535 were excluded; 406 presented with a suspicion of isolated DVT, and 129 were excluded due to the unavailability of the VIDAS D-dimer test. In total, 188 consecutive outpatients with a history of VTE and clinically suspected acute PE were included in the analysis. Of these patients, 38 (20%) had a concomitant DVT suspicion. Patient baseline characteristics are summarized in Table 1. The median age was 55 (IQR, 42-67) years, 86 (46%) patients were males, and 89 (47%) used anticoagulation. The majority of patients had only 1 confirmed previous VTE (*n* = 148 [79%]), which was mostly unprovoked. Prior VTE localizations included isolated PE (*n* = 106), isolated DVT (*n* = 43), and PE with concomitant DVT (*n* = 39). At the time of inclusion, 89 (47%) of patients were treated with therapeutic anticoagulation; 27 were treated with warfarin, 22 with low-molecular-weight heparins, 37 with rivaroxaban, and 3 with apixaban.

**Table 1:** Baseline characteristics.

Characteristic	Value (*N* = 188)
Age (y), median (IQR)	55 (42-67)
Male sex	86 (46)
Ethnicity	
White	171 (91)
Black	7 (3.7)
Asian	3 (1.6)
Hispanic	5 (2.7)
White + Black	1 (0.5)
White + Asian	1 (0.5)
Body mass index (kg/m^2^), median (IQR)	30 (25-34)
Family history of VTE	56 (30)
Active cancer	21 (11)
Age ≥ 80 y	11 (5.9)
Pregnant	3 (1.6)
Postpartum	1 (0.5)
Oral estrogen therapy	6 (3.2)
Smoking status	
Never smoked	98 (52)
Current smoker	26 (14)
History of smoking	64 (34)
No. of confirmed previous VTE	
1 event	148 (79)
2 events	33 (18)
3 events	5 (2.7)
4 events	2 (1.1)
Antiplatelet therapy	25 (13)
Therapeutic anticoagulant therapy	89 (47)
Warfarin	27 (30)
LMWH	22 (25)
Rivaroxaban	37 (42)
Apixaban	3 (3.4)
D-dimer level (ng/mL), median (IQR)	779 (345-2136)

Data are presented as *n* (%), unless stated otherwise.

LMWH, low-molecular-weight heparin; VTE, venous thromboembolism.

### 3.2 Overall results

Overall, PE was confirmed in 58 patients at initial testing (incidence, 31%; 95% CI, 25%-38%). Of these patients, 41 (71%) had isolated PE and 17 (29%) had PE with concomitant DVT of the lower extremities. Among the 99 patients not receiving anticoagulant treatment at initial testing, 47 were diagnosed with PE (incidence, 47%; 95% CI, 38%-57%).

During the 3-month follow-up period, 3 of the 125 patients in whom VTE was initially ruled out (with or without imaging) developed a VTE (incidence, 2.4%; 95% CI, 0.8%-6.8%). All 3 VTE cases were diagnosed during the course of anticoagulation therapy (breakthrough events) and involved patients with active cancer; 1 case was an isolated PE, and 2 were isolated DVTs of the lower extremities. Two patients, 1 of whom was not on anticoagulation at initial testing, were lost-to-follow-up, and 3 patients, 2 of whom were not on anticoagulation at initial testing, died. All deaths were adjudicated as unlikely due to VTE.

### 3.3 Diagnostic strategies

#### 3.2.1 YEARS algorithm

Overall, 57 patients (30%) had a low C-PTP and 131 patients (70%) had a high C-PTP. Of the 87 patients (46%) in whom PE would have been excluded without the use of imaging based on a negative YEARS strategy, 2 patients (2.3%; 95% CI, 0.6%-8.0%) were diagnosed with PE because they had been subjected to imaging tests for suspected PE at initial testing according to the locally applied diagnostic strategy (Table 2). Their VIDAS D-dimer levels were 783 and 990 ng/mL, respectively. Two patients, 1 of whom without anticoagulation at baseline, were lost-to-follow-up. Both patients were in the subgroup with a negative YEARS strategy. All 3 patients who developed VTE during the follow-up period, were in the subgroup with a positive YEARS strategy. Therefore, the overall diagnostic miss rate of the YEARS strategy would have been 2.4% (2/83; 95% CI, 0.7%-8.4%) and 6.9% (2/29; 95% CI,: 1.9%-22%) in the subgroup of patients not on anticoagulant therapy at initial testing. The sensitivity and specificity were 97% (95% CI, 88%-99%) and 65% (95% CI, 57%-73%), respectively. The PPV and NPV were 55% (95% CI, 46%-65%) and 98% (95% CI, 92%-99%), respectively (Table 3).

**Table 2:** Occurrence of recurrent pulmonary embolism and venous thromboembolism at initial testing and during the 3-mo follow-up, stratified according to the YEARS algorithm.

At baseline					
YEARS algorithm^a^	All patients	Patients managed with imaging^b^	Isolated PE at baseline	PE + DVT at baseline	Total PE incidence at baseline
Overall population	188 (100)	176 (94)	41 (22, 17-28)	17 (9.0, 5.7-14)	58 (31, 25-38)
Low C-PTP + negative D-dimer test	41 (22)	32 (78)	2 (4.9, 1.4-16)	0 (0.0, 0.0-8.6)	2 (4.9, 1.4-16)
High C-PTP + negative D-dimer test	46 (24)	45 (98)	0 (0.0, 0.0-7.7)	0 (0.0, 0.0-7.7)	0 (0.0, 0.0-7.7)
Low C-PTP + positive D-dimer test	16 (8.5)	15 (94)	6 (38, 18-61)	3 (19, 6.6-43)	9 (56, 33-77)
High C-PTP + positive D-dimer test	85 (45)^c^	84 (99)	33 (39, 29-49)	14 (16, 10-26)	47 (55, 45-65)
Patients without indication for imaging	87 (46)	77 (89)	2 (2.3, 0.6-8.0)	0 (0.0, 0.0-4.2)	2 (2.3, 0.6-8.0)

During the 3-mo follow-up					
YEARS algorithm^a^	No. of patients^d^		VTE events during follow-up		
	Overall	Without anticoagulation at baseline	Overall	At confirmation on anticoagulation	At confirmation not on anticoagulation
Overall population	125 (100)	49 (39)	3 (2.4, 0.8-6.8)	3 (2.4)	0 (0.0)
Low C-PTP + negative D-dimer test	39 (31)	15 (38)	0 (0.0, 0.0-9.0)	0 (0.0)	0 (0.0)
High C-PTP + negative D-dimer test^e^	44 (35)	14 (32)	0 (0.0, 0.0-8.0)	0 (0.0)	0 (0.0)
Low C-PTP + positive D-dimer test	7 (5.6)	5 (71)	0 (0.0, 0.0-35)	0 (0.0)	0 (0.0)
High C-PTP + positive D-dimer test^f^	35 (28)	15 (43)	3 (8.6, 3.0-22)^g^	3 (8.6)^g^	0 (0.0)
Patients without indication for imaging^e^	83 (66)	29 (35)	0 (0.0, 0.0-4.4)	0 (0.0)	0 (0.0)

Data are presented as *n* (%) or *n* (%, 95% CI).

C-PTP, clinical pretest probability; DVT, deep vein thrombosis; PE, pulmonary embolism.

a Nonhigh C-PTP: no YEARS items; high C-PTP: 1-3 YEARS items; negative D-dimer test: a D-dimer result below the probability-adjusted D-dimer threshold (<1000 and <500 ng/mL for low C-PTP and high C-PTP, respectively); positive D-dimer test: a D-dimer result greater than or equal to the probability-adjusted D-dimer threshold (1000 and 500 ng/mL for low C-PTP and high C-PTP, respectively).

b Imaging modalities: computed tomography pulmonary angiography, ventilation–perfusion scan or both.

c Three patients were diagnosed with isolated DVT.

d Patients who were lost to follow-up were excluded; patients who died during follow-up were included. All deaths were adjudicated as unlikely due to VTE.

e Two patients, 1 of whom without anticoagulation at initial testing, were lost to follow-up.

f Three patients, 2 of whom without anticoagulation at initial testing, died.

g One patient was diagnosed with recurrent PE while on full-dose low-molecular-weight heparin (LMWH). Two patients were diagnosed with recurrent DVT while on LMWH: intermediate dose (*n* = 1) and full dose (*n* = 1). Their D-dimer levels were 886, 1284, and 2285 ng/mL, respectively.

**Table 3:** Diagnostic performance of the YEARS algorithm and PEGeD algorithm to rule out pulmonary embolism at initial testing

Performance measure	YEARS algorithm	PEGeD algorithm
Diagnostic miss rate	2/87 (2.3 [0.6-8.0])	2/80 (2.5 [0.7-8.7])
Sensitivity	56/58 (97 [88-99])	56/58 (97 [88-99])
Specificity	85/130 (65 [57-73])	78/130 (60 [51-68])
PPV	56/101 (55 [46-65])	56/108 (52 [43-61])
NPV	85/87 (98 [92-99])	78/80 (98 [91-99])

Data are presented as numerator/denominator (% [95% CI]).

NPV, negative predictive value; PEGeD, Pulmonary Embolism Graduated D-dimer; PPV, positive predictive value.

#### 3.2.2 PEGeD algorithm

A total of 62 patients (33%) had a low C-PTP, 95 patients (51%) had a moderate C-PTP and 31 patients (16%) a high C-PTP based on the Wells-score. Of the 80 patients (43%) in whom PE would have been excluded without the use of imaging on the basis of a negative PEGeD strategy, 2 patients (2.5%; 95% CI, 0.7%-8.7%) were diagnosed with PE at initial testing (Table 4). Both patients had a low C-PTP and a D-dimer level of <1000 ng/mL but had been subjected to imaging tests for suspected PE at baseline showing PE. Two patients were lost to follow-up: 1 in the group with a moderate C-PTP and a D-dimer level of <500 ng/mL (ie, negative PEGeD strategy) who was not on anticoagulant therapy at initial testing, and 1 with a high C-PTP. Of the 3 patients who developed VTE during the follow-up period, 1 was in the group with a moderate C-PTP and a D-dimer level of ≥500 ng/mL, and 2 in the group with a high C-PTP. Therefore, the overall diagnostic miss rate of the PEGeD strategy would have been 2.6% (2/77; 95% CI, 0.7%-9.0%) and 7.7% (2/26; 95% CI, 2.1%-24%) in the subgroup of patients without anticoagulation at initial testing. The sensitivity and specificity were 97% (95% CI, 88%-99%) and 60% (95% CI, 51%-68%), respectively. The PPV and NPV were 52% (95% CI, 43%-61%) and 98% (95% CI, 91%-99%), respectively (Table 3).

**Table 4:** Occurrence of recurrent pulmonary embolism and venous thromboembolism at initial testing and during the 3-mo follow-up, stratified according to the PEGeD algorithm.

At baseline					
PEGED algorithm^a^	All patients	Patients managed with imaging^b^	Isolated PE at baseline	PE + DVT at baseline	Total PE incidence at baseline
Overall population	188 (100)	176 (94)	41 (22, 17-28)	17 (9.0, 5.7-14)	58 (31, 2.5-38)
Low C-PTP + negative D-dimer test	43 (23)	34 (79)	2 (4.7, 1.3-15)	0 (0.0, 0.0-8.2)	2 (4.7, 1.3-15)
Moderate C-PTP + negative D-dimer test	37 (20)	36 (97)	0 (0.0, 0.0-9.4)	0 (0.0, 0.0-9.4)	0 (0.0, 0.0-9.4)
Low C-PTP + positive D-dimer test	19 (10)	18 (95)	7 (37, 19-59)	4 (21, 8.5-43)	11 (58, 36-77)
Moderate C-PTP + positive D-dimer test	58 (31)^c^	58 (100)	22 (38, 27-51)	9 (16, 8.4-27)	31 (53, 41-66)
High C-PTP	31 (16)^d^	30 (97)	10 (32, 19-50)	4 (13, 5.1-29)	14 (45, 29-62)
Patients without indication for imaging	80 (43)	70 (88)	2 (2.5, 0.7-8.7)	0.0 (0.0, 0.0-4.6)	2 (2.5, 0.7-8.7)

During the 3-mo follow-up					
PEGED algorithm^a^	No. of patients^e^		VTE events during follow-up		
	Overall	Without anticoagulation at baseline	Overall	At confirmation on anticoagulation	At confirmation not on anticoagulation
Overall population	125 (100)	49 (39)	3 (2.4, 0.8-6.8)	3 (2.4)	0 (0.0)
Low C-PTP + negative D-dimer test	41 (33)	15 (37)	0 (0.0, 0.0-8.6)	0 (0.0)	0 (0.0)
Moderate C-PTP + negative D-dimer test^f^	36 (29)	11 (31)	0 (0.0, 0.0-9.6)	0 (0.0)	0 (0.0)
Low C-PTP + positive D-dimer test	8 (6.4)	6 (75)	0 (0.0, 0.0-32)	0 (0.0)	0 (0.0)
Moderate C-PTP + positive D-dimer test^g^	26 (21)	12 (46)	1 (3.9, 0.7-19)^h^	1 (3.9)^h^	0 (0.0)
High C-PTP^i^	14 (11)	5 (36)	2 (14, 4.0-40)^j^	2 (14)^j^	0 (0.0)
Patients without indication for imaging^f^	77 (62)	26 (34)	0 (0.0, 0.0-4.8)	0 (0.0)	0 (0.0)

Data are presented as *n* (%) or *n* (%, 95% CI).

C-PTP, clinical pre-test probability; DVT, deep vein thrombosis; LMWH, low-molecular-weight heparin; PE, pulmonary embolism; PEGeD, Pulmonary Embolism Graduated D-dimer.

a Low C-PTP: Wells 0-4 points; moderate C-PTP: Wells 4.5-6 points; high C-PTP Wells ≥ 6.5 points; negative D-dimer test: a D-dimer result below the probability-adjusted D-dimer threshold (<1000 and <500 ng/mL for low C-PTP and moderate C-PTP, respectively); positive D-dimer test: a D-dimer result greater than or equal to the probability-adjusted D-dimer threshold (1000 and 500 ng/mL for low C-PTP and moderate C-PTP, respectively).

b Imaging modalities: computed tomography pulmonary angiography, ventilation–perfusion scan or both.

c One patient was diagnosed with isolated DVT.

d Two patients were diagnosed with isolated DVT.

e Patients who were lost to follow-up were excluded; patients who died during follow-up were included. All deaths were adjudicated as unlikely due to VTE.

f One patient, without anticoagulation at baseline, was lost to follow-up.

g One patient, without anticoagulation at baseline, died.

h One patient was diagnosed with recurrent DVT while on intermediate-dose LMWH.

i One patient, without anticoagulation at baseline, was lost to follow-up. Two patients, of whom 1 without anticoagulation at baseline, died.

## 4 Discussion

Overall, both diagnostic algorithms showed comparable safety and efficiency. Our findings indicate that the use of these novel diagnostic strategies can lead to a reduction of diagnostic imaging tests by at least 43% in patients with a history of VTE suspected of a new episode of acute PE. However, the diagnostic miss rate was high and exceeds the threshold of what is considered safe in the international literature, particularly in those not receiving anticoagulant therapy [23]. Notably, although we performed a prospective diagnostic management study, not all patients were managed according to either of the 2 algorithms, and many patients received imaging tests even though these were not indicated. Had a fixed D-dimer threshold of 500 ng/mL or an age-adjusted threshold been applied, the 2 PE diagnoses missed at baseline would have been detected.

There is a lack of diagnostic studies specifically focused on patients with a history of VTE suspected of PE. To date, only 1 prospective study has evaluated the diagnostic performance of a CDR for suspected PE in patients with previous VTE [17]. In this clinical outcome study, 516 inpatients and outpatients with suspected recurrent acute PE underwent a diagnostic workup based on the modified Wells rule, D-dimer testing, and CTPA. CTPA was safely avoided in 17% of patients; none of them were diagnosed with VTE after 3 months (0%; 95% CI, 0%-3.4%). A meta-analysis that included data of 3 additional studies with a total of 1286 patients with clinically suspected acute PE and a history of VTE reported that, although fewer patients could have been managed without CTPA compared with those with a first suspected PE (15% vs 30%), the 3-month failure rate was low and similar to that of patients with a first PE suspicion (0.8%; 95% CI, 0.1%-2.4%) [24].

Consistent with these findings, results from an individual-patient data meta-analysis (IPD-MA) involving 1116 patients with prior VTE from 6 prospective studies (*N* = 6986) support the use of CDRs for ruling out PE in this specific patient subgroup with prior VTE [25]. The associated 3-month failure rate of the dichotomized Wells rule was 1.3% (95% CI, 0.1%-13%) when the conventional fixed D-dimer threshold was applied and 1.2% (95% CI, 0.1%-12%) when the age-adjusted D-dimer threshold was applied. The application of the age-adjusted D-dimer threshold led to a small increase in the proportion of patients who could be managed without imaging (33% vs 28%).

Notably, only 1 study, a large IPD-MA by Stals et al. [15], evaluated the diagnostic performance of novel CDRs with probability-adjusted D-dimer thresholds along with the well-known Wells rule and revised Geneva score, followed by fixed and adapted D-dimer thresholds for suspected acute PE [15]. In this IPD-MA, which included 2941 patients with previous VTE from 16 studies (*N* = 20,553), the failure rate of the YEARS and PEGeD algorithms was found to be higher than that of other diagnostic strategies. On the contrary, these novel CDRs performed better from an efficiency point of view. The strategies were associated with a failure rate exceeding the 2.0% margin recommended by the International Society on Thrombosis and Haemostasis (ISTH), with a failure rate of 3.5% (95% CI, 2.3%-5.2%) and 3.4% (95% CI, 2.3%-5.2%) for the YEARS and the PEGeD (Wells) algorithms, respectively [23]. However, as in our study, the novel CDRs were applied post hoc in nearly all of the studies included, and a per-protocol analysis could not be performed. As more patients underwent imaging than was actually indicated by the respective diagnostic strategies, it is likely that a greater number of isolated subsegmental PE cases were detected that might otherwise have gone undetected. Unfortunately, data on the location of PEs were unavailable in this IPD-MA dataset. Importantly, all diagnostic failures in our analysis involved segmental or more proximally localized PE and were detected during initial imaging tests, which were not actually indicated by the YEARS or PEGeD algorithm. For this reason, our findings do not provide definitive evidence that these algorithms can be used. Nevertheless, in our view, it is still too premature to conclude that the YEARS and PEGeD algorithm should not be used routinely in the diagnostic management of patients with prior VTE, mainly due to the low sample size, leaving still an unmet clinical need. Notably, patients with prior VTE were implicitly part of the prospective diagnostic outcome studies that validated the YEARS and PEGeD algorithm, and it can be argued that these patients should be approached differently than those with a first suspected VTE event based on current evidence. Further research, ideally a randomized study, is necessary to provide definitive answers regarding the safety of these CDRs in the context of suspected acute PE in patients with a history of VTE.

Our study has strengths and limitations. Its main strength lies in the multicenter design, which reflects daily clinical practice. Moreover, all VTE recurrences and deaths were adjudicated by an independent committee, which reinforces the reliability of our results, as diagnosing VTE recurrence is challenging. Still, important limitations need to be discussed. First and most important is that the YEARS and PEGeD strategies were applied post hoc. Therefore, more patients received imaging than would have been the case when these diagnostic strategies were applied in a prospective manner, potentially resulting in an overestimation of the diagnostic failure rate. Additionally, data on isolated subsegmental PE, which may not always require treatment, were not collected, preventing a direct comparison with the results from the IPD-MA by Stals et al. [15]. Another limitation is the exclusion of a large number of patients due to the use of standard-of-care D-dimer assays that provided only binary results (positive or negative) rather than continuous D-dimer levels. This limitation prevented the application of C-PTP–adjusted D-dimer thresholds in many study patients, and resulted in a small sample size for this secondary analysis of the original PREDICTORS study.

## 5 Conclusion

In this preplanned secondary analysis of the PREDICTORS study, the YEARS and PEGeD algorithms demonstrated similar safety and efficiency in patients with prior VTE suspected of having acute PE. However, the observed diagnostic miss rates were higher than expected and exceeded the safety margin as defined by the ISTH. Nevertheless, in our view, it is still premature to conclude that these CDRs are inappropriate for use in the diagnostic management of these patients. A formal, preferably randomized, diagnostic management study is necessary to provide definitive answers regarding the safety of these CDRs in the context of suspected recurrent PE.
