# Supplementary material for: The diagnostic performance of the YEARS and Pulmonary Embolism Graduated D-dimer algorithms in patients with prior venous thrombosis suspected of pulmonary embolism
Source: Res Pract Thromb Haemost. 2026 Apr 30;10(4):106617. doi: 10.1016/j.rpth.2026.106617 (PMC13264360; doi:10.1016/j.rpth.2026.106617)
Supplement: Supplementary Figures S1 and S2 [file mmc1.docx]

**The diagnostic performance of the YEARS and PEGeD algorithms in patients with prior venous thrombosis suspected of pulmonary embolism**

**Authors’ names:**
E.S.L. Martens^1^, V. Mai^2^, V. Bates^2^, A. Delluc^2^, P. Girard^3^, M.V. Huisman^1^, S.R. Kahn^4^, C. Kearon^5^, M.J. Kovacs^6^, A. Pecarskie^2^, M. Righini^7^, M. Rodger^8^, D. Scarvelis^2^, S. Schulman^5^, S. Shivakumar^9^, M. Tan^10^, V. Thiruganasambandamoorthy^11^, S. Visser^12^, P.S. Wells^2^, G. Le Gal^2^, F.A. Klok^1^ – for the PREDICTORS study group

**Affiliations:** ^1^ Department of Medicine – Division of Thrombosis and Hemostasis, Leiden University Medical Center, Leiden, the Netherlands.

^2^ Department of Medicine, Ottawa Hospital Research Institute, University of Ottawa, Ottawa, Canada.

^3^ Département de Pneumologie, Institut Mutualiste Montsouris, Paris, France; French Clinical Research Infrastructure Network INvestigation Network On Venous Thrombo-Embolism Network, Saint-Etienne, France.

^4^ Division of Internal Medicine, Department of Medicine, McGill University, Montreal, Quebec, Canada.

^5^ Department of Medicine and Thrombosis and Atherosclerosis Research Institute, McMaster University, Hamilton, Ontario, Canada.

^6^ Department of Medicine - Division of Hematology, University of Western Ontario, London, Ontario, Canada.

^7^ Division of Angiology and Hemostasis, Geneva University Hospitals and Faculty of Medicine, Geneva, Switzerland.

^8^ Department of Medicine, McGill University, McGill University Health Center, Montreal, Quebec, Canada.

^9^ Department of Medicine, Dalhousie University, Nova Scotia Health, Halifax, Nova Scotia, Canada.

^10^ Department of Medicine, Hospital Gelderse Vallei, Ede, The Netherlands..

^11^ Department of Emergency Medicine, University of Ottawa, Ottawa, Ontario, Canada.

^12^ Department of Emergency Medicine, Hôpital Montfort, Ottawa, Ontario, Canada.

**Corresponding author:** Frederikus A. Klok, MD, FESC; Department of Medicine – Thrombosis and Hemostasis, Leiden University Medical Centre, Leiden, the Netherlands; Phone: +31-71-529 8127; E-mail: [f.a.klok@LUMC.nl](mailto:f.a.klok@LUMC.nl)

**Keywords:** Venous thromboembolism; Pulmonary embolism; Diagnosis; Clinical decision rules

**Supplementary material**

**
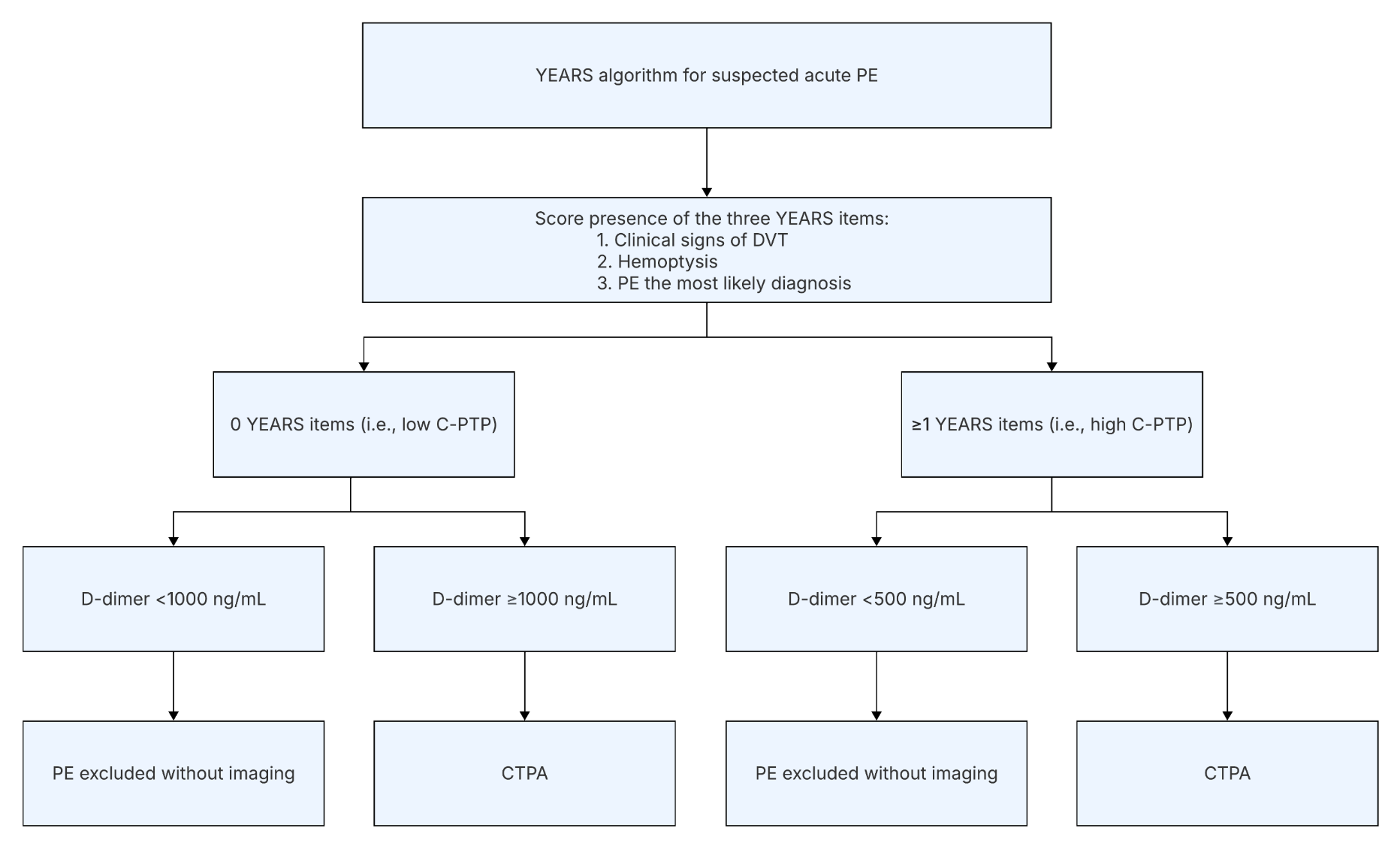
**

**Figure S1. YEARS algorithm**

Abbreviations: PE, pulmonary embolism; DVT, deep vein thrombosis; C-PTP, clinical pre-test probability; CTPA, computed tomography pulmonary angiography.

**
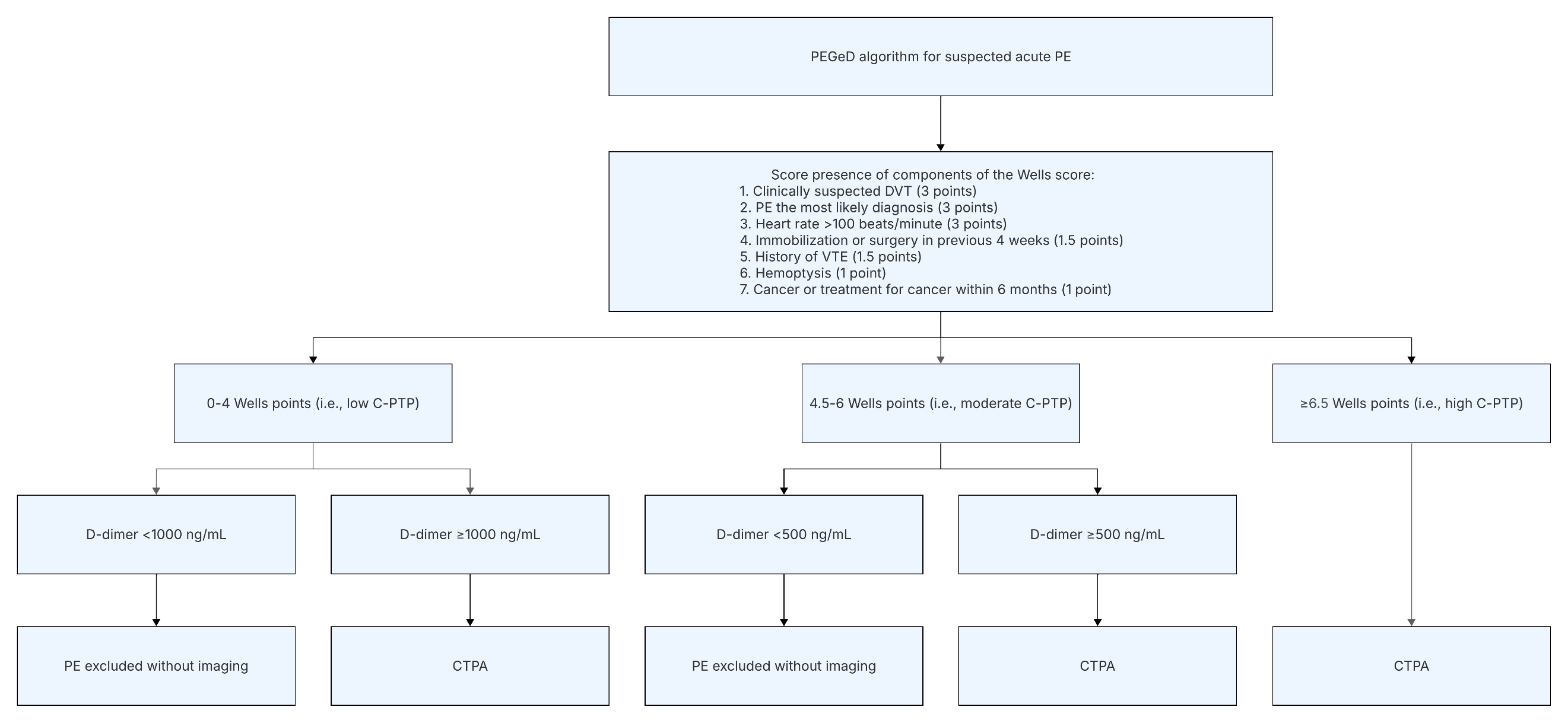
**

**Figure S2. PEGeD algorithm**

Abbreviations: PEGeD, pulmonary embolism graduated D-dimer; PE, pulmonary embolism; DVT, deep vein thrombosis; VTE, venous thromboembolism; C-PTP, clinical pre-test probability; CTPA, computed tomography pulmonary angiography.
